# A Statistical Approach to Neutron Stars’ Crust–Core Transition Density and Pressure

**DOI:** 10.3390/e25121652

**Published:** 2023-12-13

**Authors:** Ilona Bednarek, Wiesław Olchawa, Jan Sładkowski, Jacek Syska

**Affiliations:** 1 Institute of Physics, University of Silesia, 75 Pułku Piechoty 1, 41-500 Chorzów, Poland; ilona.bednarek@us.edu.pl (I.B.); jacek.syska@us.edu.pl (J.S.); 2 Institute of Physics, University of Opole, Oleska 48, 45-052 Opole, Poland; wolch@uni.opole.pl

**Keywords:** neutron star, symmetry energy, nuclear matter modeling

## Abstract

In this paper, a regression model between neutron star crust–core pressure and the symmetry energy characteristics was estimated using the Akaike information criterion and the adjusted coefficient of determination $R_{adj}^{2}$. The most probable value of the transition density, which should characterize the crust–core environment of the sought physical neutron star model, was determined based on the obtained regression function. An anti-correlation was found between this transition density and the main characteristic of the symmetry energy, i.e., its slope *L*.

## 1. Introduction

Nuclear symmetry energy is a key factor that defines the problem of the neutron star’s exact internal structure and, to some extent, determines its solution. How and in what range symmetry energy controls the emergence of different phases of nuclear matter is one of the main topics of current theoretical research in nuclear physics and astrophysics. The uncertainties in the internal structure of a neutron star, which is expected to exhibit nuclear matter at different physical states, are mainly due to the limited knowledge of the equation of state (EoS) of such a matter being in extreme physical conditions of density, temperature, and isospin asymmetry. Without experimental data extracted at such extreme conditions, it is necessary to use models that meet the results of ground-based experiments and reproduce nuclear matter’s saturation properties. Such models yield considerable uncertainty when extrapolated and applied to densities relevant to neutron stars. There are many dubious points in the modeling of neutron stars. One of the most critical concerns is the precise description of the crust–core crossing boundary and, thus, the extent of the crust. The neutron star’s matter EoS allows a neutron star’s hydrostatic model to be obtained, and its general stratification distinguishes three layers: the outermost is the atmosphere and then the crust, which splits into the inner and outer parts. The inner crust extends outward to the well-determined neutron drip density $\rho_{drip}=4\times 10^{11}$ g/cm${}^{3}$. The very inner part of a neutron star is a liquid core comprising interacting neutrons in β-equilibrium with the admixture of protons and electrons. Theoretical considerations point to the complex structure of a neutron star’s inner crust. It consists of atomic nuclei with significant neutron excess immersed in a gas of free neutrons and relativistic degenerate electrons. Depending on the density, atomic nuclei have different shapes, being spherical in most of the inner crust. Calculations suggest that non-spherical configurations of nuclei in the crust’s deepest layers become energetically favorable, forming the pasta phase [1,2,3,4,5]. This complex structure of the inner crust transforms into its equally complicated EoS. The missing precise physical model that allows for constructing an accurate EoS adequate to describe the asymmetric nuclear matter in the full range of densities characteristic of a neutron star and that correctly reproduces its properties forces the use of approximate methods. However, these methods allow for only approximately determining a neutron star model and, among other properties, the crust–core boundary’s location. Often, the physical model is described with various statistical characteristics. One of the most general is the joint probability distribution of all variables needed to describe the physical phenomenon under study. However, finding such a distribution by proposing a theoretical model is generally impossible. Therefore, the initial stage in constructing a physical model is selecting (a) regression model(s) between variables suspected of being essential in describing a physical phenomenon. Finding this regression model(s), in turn, helps capture the correct form of the physical theoretical model. Searching for different regression functions between different variables may be the initial stage of its search in the case of ignorance of the fundamental formulation of the physical model. The obvious help is appropriate statistical analysis. One of the most elegant statistical methods is the maximum likelihood method (MLM) [6] and the resulting Akaike information criterion (AIC) [7,8,9,10]. In general, the AIC helps search for the actual statistical model from which the data visible in the observation are generated. Between the models accepted for analysis, the statistical model (e.g., the regression model) closest to the unknown accurate statistical model gives the highest probability of producing the observed data. Section 4.2 is devoted to the AIC criterion, which selects a regression model between the crust–core transition pressure $P_{t}$ and the characteristics of the system’s energy. The statistics that measure the goodness of fit of the dependent variable to the data for a specific group of independent variables in a linear regression model is the coefficient of determination $R^{2}$. In this paper, the adjusted $R^{2}$, $R_{adj}^{2}$, is also used [10]; see Appendix A.1. $R_{adj}^{2}$ helps to eliminate the overestimation of the model obtained by applying $R^{2}$; i.e., $R_{adj}^{2}$ may have a maximum. It may decline as the number of regression model’s effects increases. The maximum of $R_{adj}^{2}$ indicates where the expansion of the regression model should be stopped so as not to overfit the model in the sample when compared to the unknown model in the population (theoretical model). This paper considers the $R_{adj}^{2}$ to be an auxiliary criterion in searching for the optimal regression model. Another method used in this paper for selecting the appropriate regression model is the backward elimination method [10] (Appendix A.2), which allows for choosing a regression model with factors that have a significant statistical impact on the goodness of fit of the dependent variable to the data. It is good if all these methods point to the same group of factors and produce the same regression model, although there is generally no guarantee that this will happen. In this paper, the AIC criterion for selecting the regression model is preferred, as it gives the highest probability of the appearance of the particular data. The regression model between the transition pressure $P_{t}$ and the system’s energy characteristics estimated in this paper using the AIC method is a particular characteristic of the sought actual physical model expected to describe nuclear and astrophysical observations correctly. One of the quantities characterizing a physical system is the crust–core transition density $n_{t}$. The proposed approach allows for determining the most probable value of the transition density ${\tilde{n}}_{t}$ related to the selected regression model for the analyzed sample of the RMF models (Section 5.1 and Section 5.2).

## 2. The Inner Edge of a Neutron Star Inner Crust

The location of the crust–core boundary in a neutron star can be specified if accurate models describing the matter of the crust and core are known. Generally, a hydrostatic equilibrium equation supplemented with a proper form of the EoS can provide valuable clues about the neutron star’s internal structure. However, in a neutron star’s inner crust, one can deal with a form of nuclear matter whose a priori predictions are not obvious. Model calculations indicate the possibility of a very complex, nonhomogenous phase called nuclear pasta, which further complicates the form of the equation of state of this matter. Due to its highly complex structure, determining the EoS of matter in this layer of a neutron star is problematic and burdened with very high uncertainty. Thus, it has become necessary to develop alternative methods to estimate the transition density at which homogeneous matter becomes unstable against small density fluctuations, indicating the beginning of the formation of the nucleus clusters. Below, the location of the inner boundary of the neutron star’s inner crust is determined based on thermodynamic methods [11,12,13,14], which require that the system meets the stability condition given by the pair of inequalities:

(1) $$-{\left(\frac{\partial P}{\partial v}\right)}_{\mu }>0,\;\;-{\left(\frac{\partial \mu_{asym}}{\partial q_{c}}\right)}_{v}>0.$$

Otherwise, it loses stability against small density fluctuations. In the above inequalities, *v* and $q_{c}$ are volume and charge per baryon number, *P* is the system’s total pressure, and $\mu_{asym}=\mu_{n}-\mu_{p}$ is the difference in neutrons’ and protons’ chemical potentials. The energy of nuclear matter considered in terms of binding energy (EoS) is given by the relation

(2) $$E\left(n_{b},\delta \right)=\frac{\varepsilon (n_{b},\delta )}{n_{b}}-M,$$

where the energy density $\varepsilon (n_{b},\delta )$ of the system is a function that depends on baryon density $n_{b}=n_{n}+n_{p}$ and the isospin asymmetry parameter δ; *M* is the nucleon mass. It is expected that the function $E(n_{b},\delta )$ can be represented by its Taylor series, which, under expansion to the fourth order around δ=0, takes the following form

(3) $$\begin{array}{cc} E(n_{b},\delta ) & =\sum_{n=0}^{\infty }E_{2n}\left(n_{b}\right)\delta^{2n}\\ \; & =E_{0}\left(n_{b}\right)+E_{2}\left(n_{b}\right)\delta^{2}+E_{4}\left(n_{b}\right)\delta^{4}+\ldots \;. \end{array}$$

Coefficients of the series (3) are functions of baryon density and denote the binding energy of the symmetric matter $E_{0}\left(n_{b}\right)$, the symmetry energy $E_{2}\left(n_{b}\right)\equiv E_{sym,2}\left(n_{b}\right)$, and the fourth-order symmetry energy $E_{4}\left(n_{b}\right)\equiv E_{sym,4}\left(n_{b}\right)$. The simplest case considers only the second-order term in (3), and it is known as the parabolic approximation. Using the dependence $\delta =1-2Y_{p}$, where $Y_{p}=n_{p}/n_{b}$ is the relative proton concentration, the following relation for the isospin-dependent part of the binding energy can be obtained:

$$E_{N,asym}\left(n_{b},Y_{p}\right)=E_{sym,2}\left(n_{b}\right){\left(1-2Y_{p}\right)}^{2}+E_{sym,4}\left(n_{b}\right){\left(1-2Y_{p}\right)}^{4}.$$

The energy per baryon of relativistic electrons has the form

$$E_{e}\left(n_{b}\right)=\frac{3}{4}\hbar c{\left(3\pi^{2}n_{b}\right)}^{1/3}Y_{e}^{1/3}.$$

The charge-neutrality condition demands that $Y_{e}=Y_{p}$. Thus, the total energy per baryon of the matter in the core is given by

$$E_{Tot}=E_{0}\left(n_{b}\right)+E_{N,asym}\left(n_{b},Y_{p}\right)+E_{e}\left(n_{b},Y_{p}\right).$$

The minimization of $E_{Tot}\left(n_{b},Y_{p}\right)$ with respect to $Y_{p}$ gives the β equilibrium condition

(4) $$\begin{array}{cc} \mu_{e}=\mu_{n}-\mu_{p} & =-\frac{\partial E_{Tot}\left(n_{b},Y_{p}\right)}{\partial Y_{p}}=4\left(1-2Y_{p}\right)E_{sym,2}\left(n_{b}\right)+\\ \; & +8{\left(1-2Y_{p}\right)}^{3}E_{sym,4}\left(n_{b}\right). \end{array}$$

For the chemical potential of relativistic electrons $\mu_{e}=\hbar c{\left(3\pi^{2}n_{b}\right)}^{1/3}Y_{e}^{1/3}$, the condition given above allows one to determine the equilibrium proton fraction $Y_{p}^{eq}$

(5) $$\begin{array}{cc} \hbar c{\left(3\pi^{2}n_{b}\right)}^{1/3}Y_{p}{\left(n_{b}\right)}^{1/3} & =4\left(1-2Y_{p}\left(n_{b}\right)\right)E_{sym,2}\left(n_{b}\right)\\ \; & +8{\left(1-2Y_{p}\left(n_{p}\right)\right)}^{3}E_{sym,4}\left(n_{b}\right). \end{array}$$

The condition $-{\left(\frac{\partial \mu_{asym}}{\partial q_{c}}\right)}_{v}>0$ is usually satisfied, whereas the inequality $-{\left(\frac{\partial P}{\partial v}\right)}_{\mu }>0$ can be expressed by requiring the expression $V_{ther}$ to be positive

(6) $$V_{ther}=2n_{b}\frac{\partial E(n_{b},Y_{p})}{\partial n_{b}}+n_{b}^{2}\frac{\partial^{2}E\left(n_{b},Y_{p}\right)}{\partial n_{b}^{2}}-{\left(\frac{\partial^{2}E\left(n_{b},Y_{p}\right)}{\partial n_{b}\partial Y_{p}}\right)}^{2}/\frac{\partial^{2}E\left(n_{b},Y_{p}\right)}{\partial Y_{p}^{2}},$$

where $E(n_{b},Y_{p})$ is the binding energy of nuclear matter. Solving Equations (5) and (6) allows for determining the value of the transition density $n_{t}$ and the corresponding proton concentration value $Y_{p}^{eq}\left(n_{t}\right)=Y_{t}$. Using the thermodynamic relation

(7) $$P=n_{b}^{2}\frac{\partial E(n_{b},Y_{p})}{\partial n_{b}}$$

to calculate the pressure of the n-p-e system of particles results in a total pressure that is the sum of contributions from nucleons ($P_{N}$) and electrons ($P_{e}$), $P_{Tot}=P_{N}+P_{e}$. The calculations made for the transition density $n_{t}$ and the corresponding $Y_{t}$ value can lead to the equation for the pressure at the crust–core boundary.

(8) $$\begin{array}{cc} P_{t}\left(n_{t}\right) & =n_{t}^{2}{\left.\frac{dE_{0}\left(n_{b}\right)}{dn_{b}}\right|}_{n_{t}}+n_{t}^{2}{\left(1-2Y_{t}\right)}^{2}\left({\left.\frac{dE_{sym,2}\left(n_{b}\right)}{dn_{b}}\right|}_{n_{t}}+{\left(1-2Y_{t}\right)}^{2}{\left.\frac{dE_{sym,4}\left(n_{b}\right)}{dn_{b}}\right|}_{n_{t}}\right)+\\ \; & +n_{t}Y_{t}\left(1-2Y_{t}\right)\left(E_{sym,2}\left(n_{t}\right)+2E_{sym,4}\left(n_{t}\right){\left(1-2Y_{t}\right)}^{2}\right). \end{array}$$

In general, it is expected that higher-order terms in the expansion (3) have to be included to obtain a more accurate description of the binding energy of systems with a significant value of the isospin asymmetry. In this case, an improvement in the accuracy of the obtained solution is expected. In further analysis, each function $E_{0}\left(n_{b}\right)$, $E_{sym,2}\left(n_{b}\right)$, and $E_{sym,4}\left(n_{b}\right)$ is represented by a Taylor series expansion around $n_{0}$. This procedure can be presented in the general form as

(9) $$E_{j}\left(n_{b}\right)=\sum_{i=0}^{\infty }\;C_{i}^{j}\;{\left(\frac{n_{b}-n_{0}}{3n_{0}}\right)}^{i}.$$

The index *j* distinguishes between symmetric δ=0 and asymmetric δ≠0 nuclear matter. The case of symmetric nuclear matter is denoted by j=0, and $E_{0}\left(n_{b}\right)$ means the binding energy of symmetric nuclear matter. The case j=2 corresponds to the second-order symmetry energy $E_{sym,2}\left(n_{b}\right)$ and j=4 the fourth-order symmetry energy $E_{sym,4}\left(n_{b}\right)$. The expansion coefficients

(10) $$C_{i}^{j}={\left(3n_{0}\right)}^{i}\frac{1}{i!}\frac{d^{i}E_{j}\left(n_{b}\right)}{dn_{b}^{i}}{|}_{n_{0}}$$

represent the following characteristics of nuclear matter: $C_{0}^{0}\equiv E_{0}\left(n_{0}\right)$ is the binding energy per nucleon of symmetric nuclear matter at a saturation density $n_{0}$, the nuclear matter incompressibility $C_{2}^{0}\equiv K_{0}$, $C_{0}^{2}\equiv E_{sym,2}\left(n_{0}\right)$ is the symmetry energy at the saturation density, $C_{1}^{2}\equiv L_{sym,2}$ is the second-order symmetry energy slope, $C_{2}^{2}\equiv K_{sym,2}$ is the curvature of the second-order symmetry energy, $C_{1}^{4}\equiv L_{sym,4}$ is the fourth-order symmetry energy slope, and $C_{2}^{4}\equiv K_{sym,4}$ is the curvature of the fourth-order symmetry energy. By applying the Taylor series expansions of the functions $E_{0}\left(n_{b}\right)$, $E_{sym,2}\left(n_{b}\right)$, and $E_{sym,4}\left(n_{b}\right)$, it is possible to obtain the approximate value of the pressure at the crust–core boundary

(11) $$\begin{array}{cc} P_{app}\left(n_{t}\right) & \approx \frac{n_{t}^{2}\left(n_{t}-n_{0}\right)}{9n_{0}^{2}}\left(K_{0}+K_{sym,2}\delta_{t}^{2}+K_{sym,4}\delta_{t}^{4}\right)+\\ \; & +L_{sym,2}\left(\frac{n_{t}\left(n_{t}-n_{0}\right)Y_{p}\left(n_{t}\right)\delta_{t}}{3n_{0}}+\frac{n_{t}^{2}\delta_{t}^{2}}{3n_{0}}\right)\\ \; & +n_{t}Y_{p}\left(n_{t}\right)\delta_{t}\left(E_{sym,2}+2E_{sym,4}\delta_{t}^{2}\right)+\\ \; & +L_{sym,4}\left(\frac{2n_{t}\left(n_{t}-n_{0}\right)Y_{p}\left(n_{t}\right)\delta_{t}^{3}}{3n_{0}}+\frac{n_{t}^{2}\delta_{t}^{4}}{3n_{0}}\right). \end{array}$$

In the case when the density dependence of the symmetry energy is given by the parabolic approximation, Equation (11) reduces to the following form

(12) $$\begin{array}{cc} P_{app}\left(n_{t}\right) & \approx \frac{n_{t}^{2}\left(n_{t}-n_{0}\right)}{9n_{0}^{2}}\left(K_{0}+K_{sym,2}\delta_{t}^{2}\right)+\\ \; & +L_{sym,2}\left(\frac{n_{t}\left(n_{t}-n_{0}\right)Y_{p}\left(n_{t}\right)\delta_{t}}{3n_{0}}+\frac{n_{t}^{2}\delta_{t}^{2}}{3n_{0}}\right)+n_{t}Y_{p}\left(n_{t}\right)\delta_{t}E_{sym,2}. \end{array}$$

Another approximate form of the expression defining the pressure can be obtained, assuming that δ equals 1, which leads to $Y_{p}=0$ and corresponds to the case of pure neutron matter

(13) $$\begin{array}{cc} P_{app}\left(n_{t}\right) & \approx {\left(\frac{n_{t}}{3n_{0}}\right)}^{2}\left(n_{t}-n_{0}\right)\left(K_{0}+K_{sym,2}+K_{sym,4}\right)+\\ \; & +\frac{n_{t}}{3n_{0}}n_{t}\left(L_{sym,2}+L_{sym,4}\right). \end{array}$$

The above equation reduces to a very simple form for the parabolic approximation of the symmetry energy:

(14) $$\begin{array}{ccc} P_{app}\left(n_{t}\right) & \approx & {\left(\frac{n_{t}}{3n_{0}}\right)}^{2}\left(n_{t}-n_{0}\right)\left(K_{0}+K_{sym,2}\right)+\frac{n_{t}}{3n_{0}}n_{t}L_{sym,2}. \end{array}$$

Only when the transition density reaches values equal to the saturation density $n_{0}$ does the dependence of pressure $P_{t}$ on parameters characterizing the incompressibility of nuclear matter disappear, and a straightforward relation $P_{app}\approx 1/3n_{0}L_{sym,2}$ is obtained.

## 3. Determination of the EoS

The determination of the EoS is based on the Lagrangian density function that is the sum of free baryon and meson fields part $L_{0}$ and the part $L_{int}$ describing the interaction. The individual parts are given in the following forms:

(15) $$\begin{array}{cc} L_{0} & =\bar{\psi }\left(i\gamma^{\mu }\partial_{\mu }-M\right)\psi +\frac{1}{2}\left(\partial^{\mu }\sigma \partial_{\mu }\sigma -m_{\sigma }^{2}\sigma^{2}\right)-\frac{1}{4}F^{\mu \nu }F_{\mu \nu }\\ \; & +\frac{1}{2}m_{\omega }^{2}\omega_{\mu }\omega^{\mu }-\frac{1}{4}B^{\mu \nu }B_{\mu \nu }+\frac{1}{2}m_{\rho }^{2}\;{\vec{\rho }}_{\mu }\cdot {\vec{\rho }}^{\;\mu }, \end{array}$$

where σ, $\omega_{\mu }$, and ${\vec{\rho }}_{\mu }$ represent the scalar-isoscalar σ, vector-isoscalar ω, and vector-isovector ρ meson fields, respectively, and ψ is the isodoublet nucleon field, $F_{\mu \nu }$ and $B_{\mu \nu }$ are field tensors defined as $F_{\mu \nu }=\partial_{\mu }\omega_{\nu }-\partial_{\nu }\omega_{\mu }$, and $B_{\mu \nu }=\partial_{\mu }{\vec{\rho }}_{\nu }-\partial_{\nu }{\vec{\rho }}_{\mu }$,

(16) $$\begin{array}{cc} L_{int} & =\bar{\psi }\left(g_{\sigma }\sigma -\left(g_{\omega }\omega_{\mu }+\frac{1}{2}g_{\rho }\vec{\tau }\cdot {\vec{\rho }}_{\mu }\right)\gamma^{\mu }\right)\psi -\frac{A}{3}\sigma^{3}-\frac{B}{4}\sigma^{4}+\frac{C}{4}{\left(g_{\omega }^{2}\;\omega_{\mu }\omega^{\mu }\right)}^{2}\\ \; & +g_{\sigma }g_{\omega }^{2}\;\sigma \left(\omega_{\mu }\omega^{\mu }\right)\left(\alpha_{1}+\frac{1}{2}\alpha_{1}^{\prime }g_{\sigma }\sigma \right)+g_{\sigma }\sigma g_{\rho }^{2}\left({\vec{\rho }}_{\mu }{\vec{\rho }}^{\;\mu }\right)\left(\alpha_{2}+\frac{1}{2}\alpha_{2}^{\prime }g_{\sigma }\sigma \right)+\\ \; & +\frac{1}{2}\alpha_{3}^{\prime }{\left(g_{\omega }g_{\rho }\right)}^{2}\left(\omega_{\mu }\omega^{\mu }\right)\left({\vec{\rho }}_{\mu }\;{\vec{\rho }}^{\;\mu }\right). \end{array}$$

The Lagrangian density function $L_{int}$ contains the Yukawa couplings between the nucleons and the meson and collects various nonlinear meson interaction terms. The individual coupling constants determine the strength of the meson interactions. The equations of motion derived based on the above Lagrangian density function $L=L_{0}+L_{int}$ were solved in the mean-field approximation. This approach separates meson fields into classical components and quantum fluctuations; the quantum fluctuation terms vanish, and only classical parts remain. The mean field limit, in the case of a static and a spherically symmetric system, leads to the following relations:

(17) $$\begin{array}{c} \sigma \;\to \;\langle \sigma \rangle \;\equiv \;s\\ \omega^{\mu }\;\to \;\langle \omega \rangle \;\equiv \;\langle \omega_{0}\rangle \;\delta^{\mu 0}\;\equiv \;\omega_{0}\\ {\vec{\rho }}^{\;\mu }\;\to \;\langle \rho_{3}\rangle \;\equiv \;\langle \rho_{0,3}\rangle \;\delta^{\mu 0}\;\equiv \;r_{0,3}. \end{array}$$

The mesons are coupled to the nucleon sources, which are also replaced by their expectation values in the mean-field ground state. The solution to the equations of motion allows one to calculate the energy density of the system

(18) $$\begin{array}{cc} \varepsilon & =\frac{1}{2}m_{\sigma }^{2}s^{2}+\frac{A}{3}s^{3}+\frac{B}{4}s^{4}-\frac{1}{2}m_{\omega }^{2}\omega_{0}^{2}-\frac{C}{4}{\left(g_{\omega }^{2}\omega_{0}^{2}\right)}^{2}+g_{\omega }\omega_{0}n_{b}-\frac{1}{2}m_{\rho }^{2}r_{0,3}^{2}+g_{\rho }r_{0,3}n_{3b}\\ \; & -g_{\sigma }s{\left(g_{\omega }\omega_{0}\right)}^{2}\left(\alpha_{1}+\frac{1}{2}\alpha_{1}^{\prime }g_{\sigma }s\right)-g_{\sigma }s{\left(g_{\rho }r_{0,3}\right)}^{2}\left(\alpha_{2}+\frac{1}{2}\alpha_{2}^{\prime }g_{\sigma }s\right)-\frac{1}{2}\alpha_{3}^{\prime }{\left(g_{\omega }\omega_{0}\right)}^{2}{\left(g_{\rho }r_{0,3}\right)}^{2}\\ \; & +\sum_{j=n,p}\frac{g}{2\pi^{2}}\int_{0}^{k_{Fj}}k^{2}\sqrt{k^{2}+M_{\mathrm{eff},j}^{2}}dk, \end{array}$$

where $M_{\mathrm{eff}}=M-g_{\sigma }s$ denotes the effective nucleon mass and $n_{3b}=\langle \bar{\psi }\gamma^{0}\tau_{3}\psi \rangle =n_{p}-n_{n}$, and *g* represents the number of degrees of freedom. The nonlinear meson interaction terms necessary for constructing a correct nuclear matter EoS alter both the isoscalar and isovector sectors [15,16]. The calculations were carried out in the framework of relativistic mean field (RMF) theory. This approach considers the nuclear many-body problem a relativistic system of baryons and mesons. In the original Walecka model, only scalar-isoscalar σ (attractive) and vector-isoscalar ω (repulsive) mesons [17,18] were involved in accounting for the saturation properties of symmetric nuclear matter. This model was then extended with the vector-isovector meson ρ and subjected to further modifications, leading to more sophisticated models containing various nonlinear self and mixed meson interaction terms [19]. Specifying this model in such an extended form allows one to successfully reproduce some ground-state properties of finite nuclei and nuclear matter. The implemented modifications increase the usefulness of the models in satisfactory descriptions of the properties of asymmetric nuclear matter [20,21]. The properties of nuclear matter determined based on RMF models rely on selected groups of parameters that are the research subject presented in papers [21,22]. The acceptance of a given parameterization depends on the degree of compliance of the determined properties of symmetric and asymmetric nuclear matter with the constraints resulting from the analysis of experimental data. The choice of experimental constraints in the case of symmetrical matter (δ=0) considers the nuclear matter’s incompressibility at saturation density $K_{0}$ in the range of 190–270 MeV [23,24,25], the skewness coefficient is Q in the range 200–1200 MeV [26], and the pressure $P(n_{b})$ is in density ranges of $\left(2n_{0},5n_{0}\right)$ and $\left(1.5n_{0},2.5n_{0}\right)$ [27,28]. Considering the asymmetric nuclear matter [29], experimental constraints apply to the coefficients characterizing the density dependence of the symmetry energy. One can specify the following limitation ranges: symmetry energy coefficient $E_{\mathrm{sym}}\left(n_{0}\right)$ − (25–35 MeV) and (30–35 MeV) [30], symmetry energy slope $L_{0}$ calculated at $n_{0}$ − (25–115 MeV) [31,32], volume part of isospin incompressibility $K_{\tau ,v}^{0}$ at $n_{0}$ − (−700–−400 MeV) [21,33,34], and the ratio of the symmetry energy in $n_{0}/2$ to its value in $n_{0}$ − (0.57–0.86) [35].

The RMF models applied in the analysis performed in this paper can be characterized and distinguished by different types of nonlinear couplings between mesons. It becomes possible to divide all models into three groups. Group I includes the BSR [36] and FSUGZ03, FSUGZ06 [37] models with the following types of mixed meson couplings: σ− $\omega^{2}$, $\sigma^{2}-\omega^{2}$, $\sigma -\rho^{2}$, $\sigma^{2}-\rho^{2}$, $\omega^{2}-\rho^{2}$. Group II of the BKA [38], G2 [39] and G$2^{★}$[40] models includes the $\sigma -\omega^{2}$, $\sigma^{2}-\omega^{2}$, $\sigma -\rho^{2}$ non-linear terms. Group III FSUGold [16], FSUGold4 [41], IU FSU, XS [42] and TM1 [43] is characterized by $\omega^{2}-\rho^{2}$. The values of parameters for individual models and saturation properties of symmetric and asymmetric nuclear matter are collected in the papers [44,45]. The energy density of the system given by Equation (18) encodes the correct form of the symmetry energy.

## 4. Regression Analysis

Various concepts that belong to the category of measuring the goodness of fit of the quality of statistical modeling have been developed, including $R^{2}$, adjusted $R^{2}$, which represents some attempt to adjust for the number of parameters in the model, AIC, and statistical backward elimination. The necessary information on the AIC method used in the paper to select the regression model is presented below. The basic information on other methods is given in the Appendix A. The approaches are not always equivalent, and using different methods allows for a better understanding of which factors in the regression models are the most important.

### 4.1. The Consistency Assumption for Considered Models

This paper assumes that every theoretical point in the sample of N=23 models is estimated consistently, that is, without any bias, at least asymptotically. Therefore, every theoretical point on the scatter diagram coincides with the estimate obtained for *n* of hypothetical experiments testing this model. It follows that in the limits n→∞ and for all the population of models, the finite sample error $\hat{E}$ tends to *E*. Therefore, the requirement to use the method is the assumption that it is possible to determine the values of the estimators’ model parameters from the experiment. Each model introduced into the analysis satisfies as many experimental constraints as possible. This group is an optimal sample of models in this paper.

### 4.2. The Akaike Information Criterion Analysis

The Akaike information criterion (AIC) [9] is very useful in mining the most probable appearance of the observed sample with the simultaneous limiting model extensions. Let the data $y=(y_{1},y_{2},\ldots ,y_{N})$ be generated by the true but unknown regression model *g* for the random variable *Y* (to simplify the notation, only the values $y_{i}$ of the response variable Y are written). Consider a regression model $f\equiv f(Y,A_{k})$ with a vector parameter $A_{k}$ as a candidate for describing the investigated interdependence between the dependent variable *Y* and the group of factors. $A_{k}$ is a free parameter of the regression model *f* as all its components $\alpha_{1}$, $\alpha_{2}$, …, $\alpha_{k}$ can be made zero in the null hypothesis (A3) (Appendix A). To select a better regression model *f* for the response variable *Y* and explanatory variables $X_{1},X_{2},\;\ldots ,\;X_{k}$ with a parameter $A_{k}$, the following form of AIC is used

(19) $$\begin{array}{c} AIC\left(f,A_{k}\right)=-2\;\ln L\left({\hat{A}}_{k}\right)+2\;\left(k+1\right)\;. \end{array}$$

Here, $L\left(A_{k}\right)\equiv L\left(y|A_{k}\right)$ denotes the likelihood function corresponding to the model *f* for a *N*-dimensional sample, ${\hat{A}}_{k}$ is a maximum likelihood method (MLM) estimator of the parameter $A_{k}$, and k+1 is the number of the estimated structural parameters in the regression model, i.e., the vector of slope coefficients $A_{k}$ = $\left(\alpha_{1},\alpha_{2},\ldots ,\alpha_{k}\right)$ plus the intercept $\alpha_{0}$. The mean of the maximization of the log-likelihood function $\ln L(y|A_{k})$ is equivalent to maximizing the expectation value $E_{g}\left[\ln f(Y,A_{k})\right]$ calculated for the true model *g* [9]. As the unknown parameter $A_{k}$ is replaced by its MLM estimator ${\hat{A}}_{k}$, thus, instead of $E_{g}\left[\ln f(Y,A_{k})\right]$, the expectation value $Q_{k}\equiv E_{g,\;h_{A_{k}}}\left[\ln f(Y,{\hat{A}}_{k})\right]$ is maximized, where $h_{A_{k}}$ is the distribution $h_{A_{k}}\left({\hat{A}}_{k}\right)$ of the estimator ${\hat{A}}_{k}$. The maximization of $Q_{k}$ is equivalent to the minimization of $-2NQ_{k}$, where *N* is the dimension of the sample. Because $AIC(f,A_{k})$ is approximately an unbiased estimator of $-2NQ_{k}$ [9], the model that minimizes $AIC(f,A_{k})$ is the candidate for the searched model. This can be confirmed by considering the Kullback–Leibler (K-L) distance between the models *f* and *g* [9]:

(20) $$\begin{array}{ccc} D(g,f) & = & E_{g}\left[\ln\;g(Y)\right]-E_{g}\left[\ln f(Y,A_{k})\right]\;. \end{array}$$

As $E_{g}\left[\ln\;g(Y)\right]$ is constant, the minimization of $AIC(f,A_{k})$ implies the selection of the model that minimizes the K-L distance chosen for the statistical analysis is model *f* from the unknown true model *g*. Details concerning the AIC model-selection procedure can be found in [9].

## 5. Discussion

### 5.1. The Results of the Selection of the Regression Models

The analysis performed in this paper uses a sample of the most reliable RMF models that describe nuclear matter whose high credibility follows from the fact that they meet the largest number of experimental constraints. Based on these models, nuclear matter EoSs given in terms of binding energy $E(n_{b},\delta )$ (2) were constructed. In the first step of the analysis, the function $E(n_{b},\delta )$ is approximated by its Taylor series expansion around δ=0. This leads to the separation of the symmetric $E_{0}\left(n_{b},0\right)\equiv E_{0}\left(n_{b}\right)$ and asymmetric $E_{asym}\left(n_{b},\delta \right)=E_{sym,2}\left(n_{b}\right)\delta^{2}+E_{sym,4}\left(n_{b}\right)\delta^{4}+\ldots$ parts of the EoS and allows one to consider the asymmetric part of the EoS at different levels of approximation. The coefficients of the expansion depend on baryon density. The analysis was carried out for the symmetry energy given by the parabolic approximation and for the case when the description of asymmetric matter additionally considers the fourth-order symmetry energy term. The transition pressure at the neutron star crust–core boundary following (8) decisively depends on the functions $E_{0}\left(n_{b}\right)$, $E_{sym,2}\left(n_{b}\right)$, and $E_{sym,4}\left(n_{b}\right)$. The approximate expression for the transition pressure given in terms of the defined expansion coefficients has the form given by Equation (11). All variables that enter this formula form the set of explanatory variables. In the parabolic approximation, it contains the following terms:

(21) $$\begin{array}{c} \left(K_{0},\;E_{2,\;Y\delta_{2}}\equiv E_{sym,2}\left(n_{0}\right)\;Y_{t,2}\;\delta_{t,2},\;L_{2,\;\delta_{2}^{2}}\equiv L_{sym,2}\;\delta_{t,2}^{2},\;\right.\\ \;\;\;\;\left.L_{2,\;Y\delta_{2}}\equiv L_{sym,2}\;Y_{t,2}\;\delta_{t,2},\;K_{2,\;\delta_{2}^{2}}\equiv K_{sym,2}\;\delta_{t,2}^{2}\right)\; \end{array}$$

and in the fourth-order approximation:

(22) $$\begin{array}{c} \left(K_{0},\;E_{2,\;Y\delta_{24}}\equiv E_{sym,2}\left(n_{0}\right)\;Y_{t,24}\;\delta_{t,24},\;L_{2,\;\delta_{24}^{2}}\equiv L_{sym,2}\;\delta_{t,24}^{2},\right.\\ L_{2,\;Y\delta_{24}}\equiv L_{sym,2}\;Y_{t,24}\;\delta_{t,24},\;K_{2,\;\delta_{24}^{2}}\equiv K_{sym,2}\;\delta_{t,24}^{2},\\ E_{4,\;Y\delta_{24}}\equiv E_{sym,4}\left(n_{0}\right)\;Y_{t,24}\;\delta_{t,24}^{3},\;L_{4,\;\delta_{24}^{4}}\equiv L_{sym,2}\;\delta_{t,24}^{2},\\ \left.L_{4,\;Y\delta_{24}}\equiv L_{sym,4}\;Y_{t,24}\;\delta_{t,24}^{3},\;K_{2,\;\delta_{24}^{2}}\equiv K_{sym,4}\;\delta_{t,24}^{4}\right)\;. \end{array}$$

These variables serve as input parameters in the regression analysis. The nuclear matter at the crust–core transition boundary is highly isospin-asymmetric. Thus, an additional approximation consisting of taking $\delta_{t}=1$, which corresponds to pure neutron matter, was also adopted. The description of nuclear matter was based on a selected group of RMF models. Although this is an optimal sample of models that meets many experimental constraints, none is the final true physical model, i.e., one with all the necessary components in the correct form. Since the true physical model is unknown, the search for it can start at a selected basic stage. This means providing statistical evidence for this physical model based on a regression analysis, which will reproduce the given sample of RMF models with the highest probability. The procedure of evaluating regression models, called model selection, has been applied. The selected model should be the one that provides an adequate representation of the data. However, it must be emphasized that it is not desirable that the selected model is represented by the maximal number of explanatory variables. The selection analysis identifies the explanatory variables for the selected regression model. Different selection procedures, such as the AIC method and the $R_{adj}^{2}$ and the backward elimination method, yielded the chosen regression model (Section 4.2 and Appendix A.1 and Appendix A.2). The analysis covers several cases. The first concerns approximations used to describe the symmetry energy, namely the parabolic approximation $E_{sym,2}\left(n_{b}\right)$ (Table 1) and the one that also considers the contribution from the fourth-order term $E_{sym,2}\left(n_{b}\right)+E_{sym,4}\left(n_{b}\right)$ (Table 2). In each table, the collected results of regression models for a different number of explanatory variables are given. The results for the pure neutron matter (the isospin asymmetry δ=1) obtained for the parabolic approximation are given in Table 3. In Table 4, the results for the fourth-order case are gathered.

Selection analysis indicates that when the parabolic approximation describes the symmetry energy for both considered values of the δ parameter, the minimum AIC value applies to the maximal model, meaning that the selected regression model covers the entire set of explanatory variables (21) (Table 1 and Table 3). In the case that δ≠1, a global AIC minimum appears (Table 1) for five explanatory variables denoted by $char=char_{2}\equiv \left(K_{0},E_{2,\;Y\delta_{2}},L_{2,\;\delta_{2}^{2}},L_{2,\;Y\delta_{2}},K_{2,\;\delta_{2}^{2}}\right)$. For pure neutron matter (δ=1), there are three explanatory variables $char=char_{2;\delta =1}\equiv \left(K_{0},L_{sym,2},K_{sym,2}\right)$ (Table 3). This situation changes when the symmetry energy function is the sum of $E_{sym,2}\left(n_{b}\right)$ and $E_{sym,4}\left(n_{b}\right)$. In this case, for δ≠1, a global AIC minimum appears (Table 2) for a model with six explanatory variables $char=char_{24}\equiv (K_{0},E_{2,\;Y\delta_{24}},L_{2,\;\delta_{24}^{2}},L_{2,\;Y\delta_{24}},K_{2,\;\delta_{24}^{2}},$$E_{4,\;Y\delta_{24}^{3}})$ selected from the set (22). For pure neutron matter (δ=1), there are three AIC selected explanatory variables $char=char_{24;\delta =1}\equiv \left(L_{sym,2},K_{sym,2},K_{sym,4}\right)$ (Table 4). The results obtained using the AIC method coincide with the results for $R_{adj}^{2}$ in three out of four cases, and the selected model is characterized by the maximal value of $R_{adj}^{2}$ (see Table 1, Table 2 and Table 3). An exception is for $\delta_{24}=1$ (Table 4), for which there is a minor compatibility violation in the third significant figure. However, for the AIC selected model, there still is a local maximum of $R_{adj}^{2}$.

When multiplied by the $Y_{t}$ factor, the roles of explanatory variables from sets (21) and (22) are practically negligible due to the small $Y_{t}$ value. Therefore, the explanatory variable multiplied by $Y_{t}$ is not considered for the regression analysis with only one factor. Otherwise, an artificial effect of a statistical nature may occur, suggesting a good fit to the data for a model with an insignificant variable.

**Figure 1:**
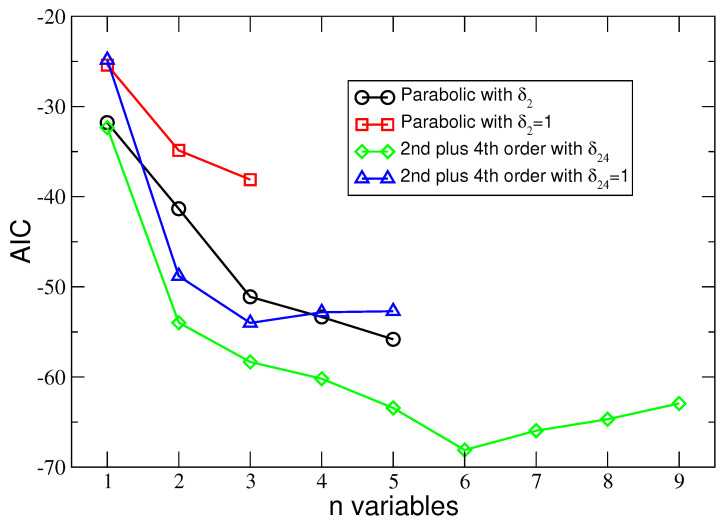
The minimal values of the AIC for a given number of explanatory variables in the regression model. The regression model with a globally minimal AIC gives the highest probability of the appearance of the sample of RMF points. The lines connecting the symbols are a guide for the eyes only.

**Figure 2:**
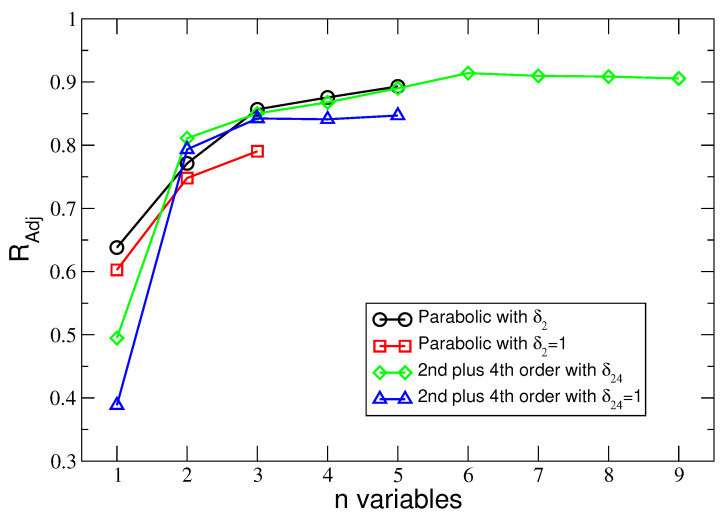
The adjusted coefficient of determination $R_{adj}^{2}$ vs the numbers n of the explanatory variables used in a given regression model. The lines connecting the symbols are a guide for the eyes only.

Results of the employed AIC and $R_{adj}^{2}$ model-selection techniques are presented in Figure 1 and Figure 2. Both figures depict values of AIC and $R_{adj}^{2}$ against the number n of explanatory variables.

### 5.2. The Most Probable Value of the Transition Density

The exact numerical values of the transition pressure $P_{t}\left(n_{t}\right)$ are calculated according to Equation (8) (see Table 5) and $P_{app}\left(n_{t}\right)$ is its approximated form given in Equation (11). The following equality is as follows:

(23) $$P_{t}\left(n_{t}\right)=P_{app}\left(n_{t}\right)+R\;,$$

where *R* is the remainder of the Taylor series expansion. This equation is valid for every RMF model from the considered sample. Treating the regression model as an alternative way to represent the data makes it possible to approximate the transition pressure in the sample with the sum of the function $P_{fit}$ plus the error (residual) term $\hat{E}$ (see Equation (A2)):

(24) $$P_{t}\left(n_{t}\right)=P_{fit}\left(char;\hat{\alpha_{j}}{|}_{j=0}^{k}\right)+\hat{E}\;,$$

where $P_{fit}\left(char;\hat{\alpha_{j}}{|}_{j=0}^{k}\right)$ is the regression function with a general form $P_{fit}\left(char,{\hat{\alpha }}_{i}\right)=\hat{\alpha_{0}}+f\left(char;\hat{\alpha_{j}}{|}_{j=1}^{k}\right)$, $\hat{\alpha_{0}}$ denotes the intercept term. The estimate of the variance of *E* is MSE, which is the variance of $\hat{E}$, and $\sqrt{MSE}$ is its standard deviation (see Appendix A.1). The regression function in the parabolic case has the form (see (21))

(25) $$\begin{array}{ccc} P_{fit} & = & {\hat{\alpha }}_{0}\;+{\hat{\alpha }}_{1}\;K_{0}+{\hat{\alpha }}_{2}\;E_{2,\;Y\delta_{2}}+{\hat{\alpha }}_{3}\;L_{2,\;\delta_{2}^{2}}+{\hat{\alpha }}_{4}\;L_{2,\;Y\delta_{2}}+{\hat{\alpha }}_{5}\;K_{2,\;\delta_{2}^{2}}\; \end{array}$$

and when the fourth-order term is included in the description of the symmetry energy, $P_{fit}$ is given by (see (22))

(26) $$\begin{array}{cc} P_{fit} & ={\hat{\alpha }}_{0}\;+{\hat{\alpha }}_{1}\;K_{0}+{\hat{\alpha }}_{2}\;E_{2,\;Y\delta_{24}}+{\hat{\alpha }}_{3}\;L_{2,\;\delta_{24}^{2}}+{\hat{\alpha }}_{4}\;L_{2,\;Y\delta_{24}}+{\hat{\alpha }}_{5}\;K_{2,\;\delta_{24}^{2}}\\ \; & +{\hat{\alpha }}_{6}\;E_{4,\;Y\delta_{24}^{3}}+{\hat{\alpha }}_{7}\;L_{4,\;\delta_{24}^{4}}+{\hat{\alpha }}_{8}\;L_{4,\;Y\delta_{24}^{3}}+\hat{\alpha_{9}}\;K_{4,\;\delta_{24}^{4}}\;. \end{array}$$

The basis for determining the most probable value of the transition density ${\tilde{n}}_{t}$ is the assumption of the validity of the relation, which is the consequence of the two possible representations of the transition pressure $P_{t}\left(n_{t}\right)$, given by Equations (23) and (24),

(27) $$P_{app}\left(n_{t}\right)=P_{fit}\left(char;\hat{\alpha_{j}}{|}_{j=0}^{k}\right)\;,$$

where the function on the RHS is given in relation (25) in the parabolic approximation or (26) in the fourth-order case. This requires the appearance of the constant $\hat{\alpha_{0}}$, which results from a different form of the coefficients in $P_{app}\left(n_{t}\right)$ and the constant coefficients $\hat{\alpha_{i}}$, i=1,2,…,k, in $P_{fit}\left(char;\hat{\alpha_{j}}{|}_{j=0}^{k}\right)$. In addition, the residual standard deviation $\sqrt{MSE}$ is a mean estimate of the remainder *R* in the range of the considered transition density $n_{t}$. The parameters of the regression model, including $\hat{\alpha_{0}}$, depend only implicitly on the transition density $n_{t}$ and in the limit $n_{t}\to 0$, ${\hat{\alpha }}_{0}\to 0.$

The regression function for the AIC and $R_{adj}^{2}$ selected regression model in the fourth-order approximation has the form

(28) $$\begin{array}{cc} P_{fit} & ={\hat{\alpha }}_{0}+{\hat{\alpha }}_{1}\;K_{0}+{\hat{\alpha }}_{2}\;E_{2,\;Y\delta_{24}}+{\hat{\alpha }}_{3}\;L_{2,\;\delta_{24}^{2}}+{\hat{\alpha }}_{4}\;L_{2,\;Y\delta_{24}}+{\hat{\alpha }}_{5}\;K_{2,\;\delta_{24}^{2}}+{\hat{\alpha }}_{6}\;E_{4,\;Y\delta_{24}^{3}}\\ \; & =-4.9549+0.002479\;K_{0}+8.009438\;E_{2,\;Y\delta_{24}}+0.08421\;L_{2,\;\delta_{24}^{2}}\\ \; & -3.4876\;L_{2,\;Y\delta_{24}}-0.004782\;K_{2,\;\delta_{24}^{2}}-49.4\;E_{4,\;Y\delta_{24}^{3}} \end{array}$$

The above regression model is also confirmed by the backward analysis procedure applied to the set of factors (22) as all values of the estimators of the structural parameters $\alpha_{j}$, j=0,1,2,…,k=6 of the regression model are statistically significant at the level α=0.05. It is assumed that the significance levels of introducing a variable into the model and keeping it in the model are the same. The other characteristics of the selected regression model with the regression function given in Equation (28) are given in Table 6. The most probable transition density $n_{t}={\tilde{n}}_{t}$ was determined by solving Equation (27). The RHS of this equation is the appropriate regression model with a specific number of factors. At the same time, the LHS in the case when the fourth-order contribution to the symmetry energy is included, following Equation (11), is expressed by elements characterizing the dependence of nuclear matter on density. Since the coefficients of the factors describing nuclear matter depend on the transition density, solving equation Equation (27) makes determining the transition density value possible. For example, the form of Equation (27) for the AIC-selected regression model is presented as

(29) $$\begin{array}{cc} \; & \frac{n_{t}^{2}\left(n_{t}-n_{0}\right)}{9n_{0}^{2}}\left(K_{0}+K_{sym,2}\delta_{t}^{2}\right)+\\ \; & +L_{sym,2}\left(\frac{n_{t}\left(n_{t}-n_{0}\right)Y_{p}\left(n_{t}\right)\delta_{t}}{3n_{0}}+\frac{n_{t}^{2}\delta_{t}^{2}}{3n_{0}}\right)\\ \; & +n_{t}Y_{p}\left(n_{t}\right)\delta_{t}\left(E_{sym,2}+2E_{sym,4}\delta_{t}^{2}\right)+\\ \; & ={\hat{\alpha }}_{0}+{\hat{\alpha }}_{1}\;K_{0}+{\hat{\alpha }}_{2}\;E_{2,\;Y\delta_{24}}+{\hat{\alpha }}_{3}\;L_{2,\;\delta_{24}^{2}}+{\hat{\alpha }}_{4}\;L_{2,\;Y\delta_{24}}+{\hat{\alpha }}_{5}\;K_{2,\;\delta_{24}^{2}}+{\hat{\alpha }}_{6}\;E_{4,\;Y\delta_{24}^{3}}\\ \; & =-4.9549+0.002479\;K_{0}+8.009438\;E_{2,\;Y\delta_{24}}+0.08421\;L_{2,\;\delta_{24}^{2}}\\ \; & -3.4876\;L_{2,\;Y\delta_{24}}-0.004782\;K_{2,\;\delta_{24}^{2}}-49.4\;E_{4,\;Y\delta_{24}^{3}}. \end{array}$$

Solving the above equation for $n_{t}$ allows one to calculate its value for the selected regression model. This procedure was carried out for each of the 23 RMF models, and then the average value $\bar{{\tilde{n}}_{t}}$ was calculated from the obtained 23 ${\tilde{n}}_{t}$ values. Similar calculations were performed for the parabolic approximation of the symmetry energy $E_{sym,2}\left(n_{b}\right)$ and the case of δ=1. The regression model selected by the AIC gives the most probable appearance of the sample [9]. Equation (27) is a relationship imposed on the model specified by the AIC that guaranteed that the observed sample appeared with maximum probability for a fixed number of factors. Thus, the value of $n_{t}={\tilde{n}}_{t}$ determined from Equation (27) is the most probable value for the determined number of factors selected by the AIC regression model. Because the AIC’s globally selected regression model is the best estimate of the true regression model, the transition density ${\tilde{n}}_{t}$ resulting from the performed regression analysis is considered the best approximation of the transition density implied by the true regression model. As a consequence, ${\tilde{n}}_{t}$ should characterize the true physical model.

In the parabolic case, the regression model selected via the AIC and $R_{adj}^{2}$ has the following regression function:

(30) $$\begin{array}{cc} P_{fit} & ={\hat{\alpha }}_{0}+{\hat{\alpha }}_{1}\;K_{0}+{\hat{\alpha }}_{2}\;E_{2,\;Y\delta_{2}}+{\hat{\alpha }}_{3}\;L_{2,\;\delta_{2}^{2}}+{\hat{\alpha }}_{4}\;L_{2,\;Y\delta_{2}}+{\hat{\alpha }}_{5}\;K_{2,\;\delta_{2}^{2}}\\ \; & =-2.9636+0.002974\;K_{0}+2.4789\;E_{2,\;Y\delta_{2}}+0.04254\;L_{2,\;\delta_{2}^{2}}\\ \; & -1.1365\;L_{2,\;Y\delta_{2}}-0.003259\;K_{2,\;\delta_{2}^{2}}\;. \end{array}$$

This regression model is confirmed through the backward analysis procedure applied to the set of factors (21) at the significance level α=0.065. The values of the estimators of the structural parameters other than $\alpha_{1}$ for the factor $K_{0}$ and $\alpha_{4}$ for the factor $L_{2,\;Y\delta_{2}}$, remain in the model at a significance level lower than α=0.05. The other characteristics of the selected regression model with the regression function given in Equation (30) are given in Table 6.

To calculate the uncertainty of the estimation of a particular value of ${\tilde{n}}_{t}$, two components, namely the error of the estimation of the conditional expectation value $P_{fit}\left(char;\alpha_{j}{|}_{j=0}^{k}\right)$ (which appeared to be decisive) and the error propagation from $P_{fit}\left(char;{\hat{\alpha }}_{j}{|}_{j=0}^{k}\right)$ to ${\tilde{n}}_{t}$, were calculated. The obtained uncertainty of the estimation of a particular ${\tilde{n}}_{t}$ coming from these two sources is, on average, approximately ±0.024 when the fourth-order symmetry energy term is included in the analysis (k=6) and ±0.03 for the parabolic case (k=5).

**Figure 3:**
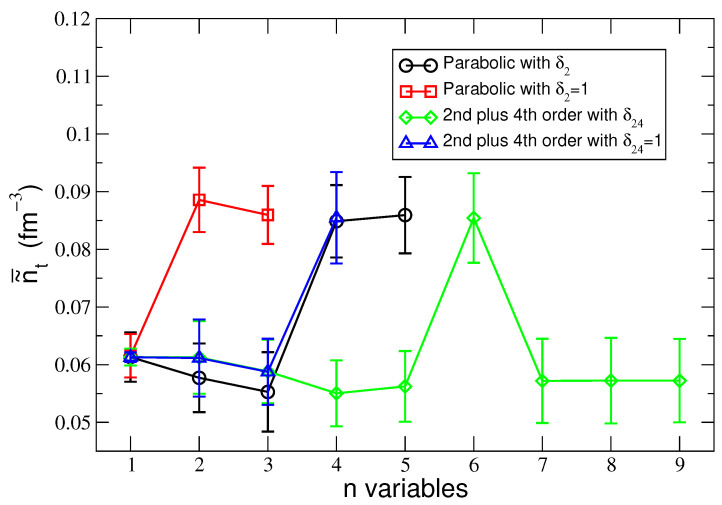
The most probable transition density $\bar{{\tilde{n}}_{t}}$ vs. the numbers n of the explanatory variables used in a given regression model selected by AIC. The maximal value of ${\tilde{n}}_{t}$ refers to the global minimal AIC. The lines connecting the symbols are a guide for the eyes only.

Figure 3 shows the values of the means $\bar{{\tilde{n}}_{t}}$ of the most probable density values ${\tilde{n}}_{t}$ (see Table 1, Table 2, Table 3 and Table 4), obtained for $\delta =\delta_{t}$ and δ=1 for the two considered cases of symmetry energy approximations as a function of the number of explanatory variables n that characterize a given regression model selected using the AIC and $R_{adj}^{2}$ methods.

**Figure 4:**
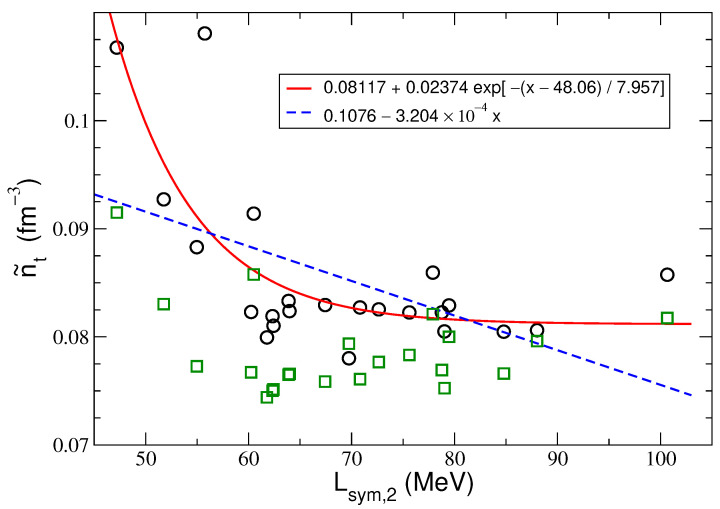
The linear and exponential fits to the sample of ($L_{sym,2};{\tilde{n}}_{t}$) points for the AIC selected model for the fourth-order case with the regression function given by Equation (28). The most probable values of ${\tilde{n}}_{t}$ (black circles) are calculated from Equations (27) and (28) for the values of $x\equiv L_{sym,2}$ for the sample of N=23 RMF models. As for the mean square errors, $MSE\left(exponential\right)=2.683\;\times 10^{-5}<MSE\left(linear\right)=4.5767\;\times 10^{-5}\;\left(fm^{-6}\right)$; thus, the exponential fit is better [50]. Green squares illustrate the $n_{t}$ values calculated for individual RMF models.

The crucial relation for the further construction of a true physical model is ${\tilde{n}}_{t}\left(L_{sym,2}\right)$. This relation for the model selected by the AIC and $R_{adj}^{2}$ in the fourth-order approximation with the regression function (28) is shown in Figure 4. Green squares illustrate the $n_{t}$ values calculated for individual RMF models. The $n_{t}$ values are shown in Table 7. The AIC method, searching for phenomena related to the location of the neutron star’s crust–core transition described by the RMF models shifts the crust–core boundary to higher densities. In the paper [14], the neutron star’s core–crust transition densities obtained within the dynamical and thermodynamical methods using the full EoS and its PA with the MDI and Skyrme interactions have been analyzed. It should be emphasized that the most probable transition density ${\tilde{n}}_{t}$ values determined in this paper, based on the proposed probabilistic method for the symmetry energy supplemented by the fourth-order contribution, given as a function of the symmetry energy slope $L_{sym,2}$, follow a very similar course as in the case of models with modified Gogny (MDI) interaction and 51 Skyrme interactions. The results reported in other papers show that the transition density $n_{t}$ decreases as *L* increases, as it is anticorrelated with *L*. This has been verified with many different models [46,47,48,49].

## 6. Conclusions

A regression model selected according to the AIC and $R_{adj}^{2}$ model-selection procedures can be used to identify and support a correct model from the physical and experimental points of view. The results obtained include, among others things, the importance of approximations used to describe the symmetry energy. Suppose the isospin asymmetry parameter has the value resulting from the adopted model $\delta =\delta_{t}$. In this case, the selected regression models corresponding to the extreme values of AIC and $R_{adj}^{2}$, both for the parabolic approximation and considering the fourth-order term, include as explanatory variables $K_{0}$ and $L_{sym,2}$, $K_{sym,2}$, and $E_{sym,2}$ multiplied by functions of $\delta_{t}$ (see (21) and (22)). The preferred regression model that incorporates contributions from the fourth-order symmetry energy term weakly depends on the fourth-order symmetry energy characteristics (depending only on $E_{sym,4}Y_{t}\delta_{24}^{3}$ ). A different result is obtained assuming the regression analysis is performed for pure neutron matter. Then, in the parabolic approximation, the set of independent variables is the maximum set and includes the factors $K_{0}$, $K_{sym,2}$, and $L_{sym,2}$. However, in the case when the symmetry energy is given by the formula $E_{sym,2}\left(n_{b}\right)+E_{sym,4}\left(n_{b}\right)$, the set of explanatory variables does not include the characteristics of symmetric nuclear matter, namely its incompressibility $K_{0}$. In this case, the regression model is described by the following independent variables: $L_{sym,2},\;L_{sym,4},\;K_{sym,2}$, and $K_{sym,4}$. Regardless of the considered values of δ, the selected regression models for the parabolic approximation always contain the maximal set of independent variables. An additional conclusion concerns the value of the most probable transition density, which for pure neutron matter, when taking into account the contribution of the fourth-order symmetry energy, is of significantly lower value $\bar{{\tilde{n}}_{t}}=0.05876\pm 0.00572$ fm${}^{-3}$. This means that, in this case, the crust–core boundary is moved to much lower densities. After obtaining the transition density in the fourth-order approximation for the model with AIC and $R_{adj}^{2}$ selected (with the regression function (28)), it is possible to examine the relationship between ${\tilde{n}}_{t}$ and $L_{sym,2}$. The obtained results confirm the existence of anti-correlation between these quantities (see Figure 4). Moreover, the mean square error MSE for the exponential fit is much lower than in the linear case. Thus, the exponential fit is better. This could suggest the existence of a valuable shift of the transition boundary towards densities approaching the saturation density in the case of nuclear matter models characterized by a low $L_{sym,2}$ value.

## Figures and Tables

**Table 1:** Some characteristics of the regression models in the parabolic approximation case with $\delta_{2}$. $R^{2}$ is the coefficient of determination, $R_{adj}^{2}$ is the adjusted coefficient of determination (Appendix A.1), AIC is the Akaike information criterion given in Equation (19), and the $\bar{{\tilde{n}}_{t}}$ values in the table are the means of the most probable density values assuming Equation (27). The most likely value is for the model with a globally minimal AIC given in boldface characters.

Variables	$R^{2}$	$R_{\mathrm{adj}}^{2}$	AIC	$\bar{{\tilde{n}}_{t}}$
$L_{2,\;Y\delta_{2}}$	0.7629	0.7516	−40.4399	
$L_{2,\;\delta_{2}^{2}}$	0.6544	0.6380	−31.7806	0.06131 ± 0.00427
$K_{2,\;\delta_{2}^{2}}$	0.1782	0.1391	−11.8554	
$E_{2,\;Y\delta_{2}}$	0.0197	−0.0270	−7.7978	
$K_{0}$	0.0001	−0.0475	−7.3445	
($L_{2,\;Y\delta_{2}}$ $K_{2,\;\delta_{2}^{2}}$)	0.8224	0.8046	−44.9683	
($E_{2,\;Y\delta_{2}}$ $L_{2,\;\delta_{2}^{2}}$)	0.7920	0.7712	−41.3372	0.05772 ± 0.00596
($E_{2,\;Y\delta_{2}}$ $L_{2,\;\delta_{2}^{2}}$ $K_{2,\;\delta_{2}^{2}}$)	0.8763	0.8567	−51.1033	0.05527 ± 0.00688
($L_{2,\;Y\delta_{2}}$ $L_{2,\;\delta_{2}^{2}}$ $K_{2,\;\delta_{2}^{2}}$)	0.8624	0.8407	−48.6563	
($K_{0}$ $E_{2,\;Y\delta_{2}}$ $L_{2,\;\delta_{2}^{2}}$ $K_{2,\;\delta_{2}^{2}}$)	0.8982	0.8756	−53.3467	0.08487 ± 0.00628
($E_{2,\;Y\delta_{2}}$ $L_{2,\;Y\delta_{2}}$ $L_{2,\;\delta_{2}^{2}}$ $K_{2,\;\delta_{2}^{2}}$)	0.8932	0.8694	−52.2390	
( $K_{0}$ $E_{2,\;Y\delta_{2}}$ $L_{2,\;Y\delta_{2}}$ $L_{2,\;\delta_{2}^{2}}$ $K_{2,\;\delta_{2}^{2}}$)	**0.9173**	**0.8930**	**−55.8201**	**0.08593** ± **0.00663**

**Table 2:** Some characteristics of the regression models when the fourth-order contribution is included with $\delta_{24}$. $R^{2}$ is the coefficient of determination, $R_{adj}^{2}$ is the adjusted coefficient of determination (Appendix A.1), AIC is the Akaike information criterion given in Equation (19), and $\bar{{\tilde{n}}_{t}}$ values in the table are the means of the most probable density values assuming Equation (27). The most likely value is for the model with a globally minimal AIC given in boldface characters.

Variables	$R^{2}$	$R_{\mathrm{adj}}^{2}$	AIC	$\bar{{\tilde{n}}_{t}}$
$L_{2,\;Y\delta_{24}}$	0.6637	0.6477	−40.6499	
$L_{2,\;\delta_{24}^{2}}$	0.5177	0.4947	−32.3577	0.06131 ± 0.00146
$K_{2,\;\delta_{24}^{2}}$	0.2235	0.1865	−21.4046	
$E_{4,\;Y\delta_{24}^{3}}$	0.0848	0.0413	−17.6258	
$L_{4,\;\delta_{24}^{4}}$	0.0129	−0.0341	−15.8845	
$K_{0}$	0.0090	−0.0382	−15.7954	
$K_{4,\;\delta_{24}^{4}}$	0.0025	−0.0450	−15.6451	
$L_{4,\;Y\delta_{24}^{3}}$	0.0015	−0.0460	−15.6214	
$E_{2,\;Y\delta_{24}}$	0.0014	−0.0461	−15.6199	
($L_{2,\;\delta_{24}^{2}}$ $K_{4,\;\delta_{24}^{4}}$)	0.8282	0.8110	−53.9753	0.06125 ± 0.0063
($L_{2,\;Y\delta_{24}}$ $K_{2,\;\delta_{24}^{2}}$)	0.7554	0.7310	−45.8542	
($L_{2,\;\delta_{24}^{2}}$ $K_{2,\;\delta_{24}^{2}}$ $K_{4,\;\delta_{24}^{4}}$)	0.8707	0.8503	−58.3342	0.05882 ± 0.0055
($E_{2,\;Y\delta_{24}}$ $L_{2,\;Y\delta_{24}}$ $K_{4,\;\delta_{24}^{4}}$)	0.8636	0.8421	−57.1077	
($E_{2,\;Y\delta_{24}}$ $L_{2,\;\delta_{24}^{2}}$ $K_{2,\;\delta_{24}^{2}}$ $K_{4,\;\delta_{24}^{4}}$)	0.8918	0.8678	−60.1982	0.05503 ± 0.00573
($L_{2,\;\delta_{24}^{2}}$ $L_{2,\;Y\delta_{24}}$ $K_{2,\;\delta_{24}^{2}}$ $K_{4,\;\delta_{24}^{4}}$)	0.8906	0.8663	−59.9435	
($E_{2,\;Y\delta_{24}}$ $L_{2,\;\delta_{24}^{2}}$ $L_{2,\;Y\delta_{24}}$ $K_{2,\;\delta_{24}^{2}}$ $E_{4,\;Y\delta_{24}^{3}}$)	0.9150	0.8901	−63.4363	0.05623 ± 0.00614
($L_{2,\;Y\delta_{24}}$ $K_{2,\;\delta_{24}^{2}}$ $L_{4,\;\delta_{24}^{4}}$ $L_{4,\;Y\delta_{24}^{3}}$ $K_{4,\;\delta_{24}^{4}}$)	0.9087	0.8819	−61.7911	
($K_{0}$ $E_{2,\;Y\delta_{24}}$ $L_{2,\;\delta_{24}^{2}}$ $L_{2,\;Y\delta_{24}}$ $K_{2,\;\delta_{24}^{2}}$ $E_{4,\;Y\delta_{24}^{3}}$)	**0.9375**	**0.9141**	**−68.1043**	**0.08543** ± **0.00776**
($E_{2,\;Y\delta_{24}}$ $L_{2,\;\delta_{24}^{2}}$ $L_{2,\;Y\delta_{24}}$ $K_{2,\;\delta_{24}^{2}}$ $E_{4,\;Y\delta_{24}^{3}}$ $L_{4,\;\delta_{24}^{4}}$)	0.9267	0.8992	−64.4407	
($K_{0}$ $E_{2,\;Y\delta_{24}}$ $L_{2,\;\delta_{24}^{2}}$ $L_{2,\;Y\delta_{24}}$ $K_{2,\;\delta_{24}^{2}}$ $E_{4,\;Y\delta_{24}^{3}}$ $L_{4,\;\delta_{24}^{4}}$)	0.9384	0.9097	−65.9606	0.05718 ± 0.0073
($K_{0}$ $E_{2,\;Y\delta_{24}}$ $L_{2,\;\delta_{24}^{2}}$ $L_{2,\;Y\delta_{24}}$ $K_{2,\;\delta_{24}^{2}}$ $E_{4,\;Y\delta_{24}^{3}}$ $L_{4,\;Y\delta_{24}^{3}}$)	0.9377	0.9086	−65.6782	
($K_{0}$ $E_{2,\;Y\delta_{24}}$ $L_{2,\;\delta_{24}^{2}}$ $L_{2,\;Y\delta_{24}}$ $K_{2,\;\delta_{24}^{2}}$ $E_{4,\;Y\delta_{24}^{3}}$ $L_{4,\;\delta_{24}^{4}}$ $L_{4,\;Y\delta_{24}^{3}}$)	0.9418	0.9086	−64.6885	0.05723 ± 0.00742
($K_{0}$ $E_{2,\;Y\delta_{24}}$ $L_{2,\;\delta_{24}^{2}}$ $L_{2,\;Y\delta_{24}}$ $K_{2,\;\delta_{24}^{2}}$ $E_{4,\;Y\delta_{24}^{3}}$ $L_{4,\;\delta_{24}^{4}}$ $K_{4,\;\delta_{24}^{4}}$)	0.9384	0.9033	−63.3813	
($K_{0}$ $E_{2,\;Y\delta_{24}}$ $L_{2,\;\delta_{24}^{2}}$ $L_{2,\;Y\delta_{24}}$ $K_{2,\;\delta_{24}^{2}}$ $E_{4,\;Y\delta_{24}^{3}}$ $L_{4,\;\delta_{24}^{4}}$ $L_{4,\;Y\delta_{24}^{3}}$ $K_{4,\;\delta_{24}^{4}}$)	0.9442	0.9056	−62.9401	0.05723 ± 0.00723

**Table 3:** The case when the parabolic approximation gives the symmetry energy. The regression models are determined for $\delta_{2}=1$. $\bar{{\tilde{n}}_{t}}$ in the table are the means of the most probable density values assuming Equation (27). The most likely value of $\bar{{\tilde{n}}_{t}}$ is the one obtained for the model with a globally minimal AIC value. The variables in this table are from set (21) in the case of $\delta_{2}=1$. The most likely value is for the model with a globally minimal AIC given in boldface characters.

Variables	$R^{2}$	$R_{\mathrm{adj}}^{2}$	AIC	$\bar{{\tilde{n}}_{t}}$
$L_{sym,2}$	0.6206	0.6026	−25.3983	0.06154 ± 0.00377
$K_{sym,2}$	0.2024	0.1644	−8.3058	
$K_{0}$	0.0018	−0.0457	−3.1471	
($K_{0}$ $L_{sym,2}$)	0.7709	0.7480	−34.8749	0.08857 ± 0.00559
($L_{sym,2}$ $K_{sym,2}$)	0.7558	0.7314	−33.4109	
($K_{0}$ $K_{sym,2}$)	0.2186	0.1405	−6.6566	
($K_{0}$ $L_{sym,2}$ $K_{sym,2}$)	**0.8190**	**0.7904**	**−38.1132**	**0.08596 ** ± **0.00503**

**Table 4:** The case when the symmetry energy is represented by the functions $E_{sym,2}\left(n_{b}\right)+E_{sym,4}\left(n_{b}\right)$ and for $\delta_{24}=1$. $\bar{{\tilde{n}}_{t}}$ denotes the means of the most probable density values assuming Equation (27). The most likely value is for the model with a globally minimal AIC value. The variables in this table are the ones from set (22) in the case of $\delta_{24}=1$. The most likely value is for the model with a globally minimal AIC given in boldface characters.

Variables	$R^{2}$	$R_{\mathrm{adj}}^{2}$	AIC	$\bar{{\tilde{n}}_{t}}$
$L_{sym,2}$	0.4163	0.3885	−24.8389	0.06129 ± 0.00081
$K_{sym,2}$	0.2614	0.2262	−19.4268	
$K_{0}$	0.0406	−0.0051	−13.411	
$K_{sym,4}$	0.0080	−0.0393	−12.6422	
$L_{sym,4}$	0.0040	−0.0434	−12.5512	
($L_{sym,2}$ $K_{sym,4}$)	0.8122	0.7934	−48.8023	0.06115 ± 0.00668
($K_{0}$ $L_{sym,2}$)	0.6648	0.6313	−35.4791	
($L_{sym,2}$ $K_{sym,2}$ $K_{sym,4}$)	0.8638	0.8423	**−54.004**	**0.05876** ± **0.00572**
($K_{0}$ $L_{sym,2}$ $K_{sym,4}$)	0.8320	0.8055	−49.1883	
($K_{0}$ $L_{sym,2}$ $K_{sym,2}$ $K_{sym,4}$)	0.8699	0.8410	−52.8228	0.08547 ± 0.00793
($L_{sym,2}$ $K_{sym,2}$ $L_{sym,4}$ $K_{sym,4}$)	0.8657	0.8359	−52.0968	
($K_{0}$ $L_{sym,2}$ $K_{sym,2}$ $L_{sym,4}$ $K_{sym,4}$)	0.8818	0.8470	−52.7082	0.05447 ± 0.00657

**Table 5:** The characteristics of the regression models selected by the AIC and $R_{adj}^{2}$ with the regression functions (30) in the parabolic case and (28) in the fourth-order case. SSR, SSE, and SSY are the sum of squares due to regression, the error sum of squares, and the total sum of squares of the response $Y\equiv P_{t}$, respectively, and SSY=SSR+SSE. MSE (which is the variance of the error term $\hat{E}$) is the mean squared error (Appendix A.1), [10]. ${\hat{\sigma }}_{{\hat{\alpha }}_{0}}$ to ${\hat{\sigma }}_{{\hat{\alpha }}_{5}}$ are the standard errors of ${\hat{\alpha }}_{0}$ to ${\hat{\alpha }}_{5}$ in the parabolic approximation case, and ${\hat{\sigma }}_{{\hat{\alpha }}_{0}}$ to ${\hat{\sigma }}_{{\hat{\alpha }}_{6}}$ are the standard errors of ${\hat{\alpha }}_{0}$ to ${\hat{\alpha }}_{6}$ in the fourth-order approximation case.

Order	${\hat{\sigma }}_{{\hat{\alpha }}_{0}}$	${\hat{\sigma }}_{{\hat{\alpha }}_{1}}$	${\hat{\sigma }}_{{\hat{\alpha }}_{2}}$	${\hat{\sigma }}_{{\hat{\alpha }}_{3}}$	${\hat{\sigma }}_{{\hat{\alpha }}_{4}}$	${\hat{\sigma }}_{{\hat{\alpha }}_{5}}$	${\hat{\sigma }}_{{\hat{\alpha }}_{6}}$	SSE	SSR	MSE
2-nd	0.7276	0.001335	0.9738	0.01369	0.573	0.0009326		0.06771	0.7514	0.003983
2-nd + 4-th	0.8433	0.001034	1.9201	0.01683	0.8438	0.000839	15.7805	0.03577	0.5369	0.002236

**Table 6:** Numerical values of the transition pressure $P_{t}$ calculated for individual models for $\delta =\delta_{t}$, i.e., for the value resulting from Equations (5) and (6) and for neutron matter (δ=1). The table contains pressure values for the parabolic approximation and the case when a fourth-order contribution is included in the description of the symmetry energy. The subscript 2 refers to the quantities calculated based on the parabolic approximation, and the subscript 24 indicates the sum of the second- and fourth-order contributions. The pressure $P_{t}$ is given in Mev/fm${}^{3}$.

Model	$P_{t,24}$ for $\delta_{t}=\delta_{24}$	$P_{t,24}$ for $\delta_{24}=1$	$P_{t,2}$ for $\delta_{t}=\delta_{2}$	$P_{t,2}$ for $\delta_{2}=1$
BSR8	0.299938	0.343839	0.292153	0.334073
BSR9	0.342497	0.385733	0.339439	0.382146
BSR10	0.428416	0.473291	0.439006	0.487483
BSR11	0.534352	0.583563	0.567858	0.627902
BSR12	0.675834	0.751728	0.721884	0.814746
BSR15	0.278423	0.316262	0.272518	0.308937
BSR16	0.306975	0.348112	0.303324	0.343495
BSR17	0.392911	0.437461	0.401624	0.448741
BSR18	0.510366	0.564637	0.536236	0.5984
BSR19	0.643445	0.708704	0.695239	0.778184
BSR20	0.698844	0.759885	0.777745	0.866059
FSUGoldGZ03	0.345584	0.388768	0.343134	0.385933
FSUGoldGZ06	0.310274	0.351434	0.307171	0.34749
BKA20	0.470379	0.519487	0.494073	0.549805
BKA22	0.468696	0.507039	0.495763	0.542082
BKA24	0.574315	0.617998	0.623478	0.682634
$G_{2}$	0.800376	0.851973	0.925128	1.01912
$G_{2}^{*}$	0.438749	0.471592	0.46765	0.507
FSUGold	0.55059	0.654896	0.528628	0.618851
FSUGold4	0.312472	0.391891	0.272145	0.330303
IU FSUGold	0.31685	0.404539	0.257654	0.315325
XS	0.206939	0.262488	0.18285	0.225486
TM1	0.674766	0.822694	0.645515	0.772463

**Table 7:** Numerical values of the transition density $n_{t}$ calculated for individual RMF models for $\delta =\delta_{t}$, i.e., for the value of δ resulting from Equations (5) and (6). The case of pure neutron matter (δ=1) is also included. The table contains transition density values for the parabolic approximation and the case when the fourth-order contribution in the description of the symmetry energy is included. The subscript 2 refers to the quantities calculated based on the parabolic approximation, and the subscript 24 indicates the sum of the second- and fourth-order contributions. The transition density $n_{t}$ is given in fm${}^{-3}$.

Model	$n_{t,24}$ for $\delta_{t}=\delta_{24}$	$n_{t,24}$ for $\delta_{24}=1$	$n_{t,2}$ for $\delta_{t}=\delta_{2}$	$n_{t,2}$ for $\delta_{2}=1$
BSR8	0.0767223	0.074953	0.0777133	0.0763169
BSR9	0.0764874	0.0745523	0.0776769	0.0761666
BSR10	0.0760831	0.074032	0.0778071	0.0762911
BSR11	0.0769332	0.0748801	0.0794208	0.0780486
BSR12	0.0820997	0.0802968	0.084933	0.0839669
BSR15	0.0744085	0.0725614	0.0754156	0.0739332
BSR16	0.0750102	0.0731778	0.0761299	0.0746899
BSR17	0.0758628	0.0739998	0.0774964	0.0761244
BSR18	0.077671	0.0758692	0.0799326	0.07876
BSR19	0.0800042	0.078217	0.0830318	0.0820617
BSR20	0.0796037	0.0777179	0.0833096	0.0823611
FSUGoldGZ03	0.0765717	0.0746572	0.0777949	0.0763086
FSUGoldGZ06	0.0751303	0.0733176	0.0762822	0.0748653
BKA20	0.0783231	0.076584	0.0804157	0.0792261
BKA22	0.0752489	0.0731542	0.0775529	0.0760526
BKA24	0.0766072	0.0744403	0.0796047	0.0781916
G2	0.0817356	0.079525	0.0865901	0.085502
${G2}^{*}$	0.0793737	0.0777159	0.0819961	0.0808773
FSUGold	0.0857725	0.0847514	0.0865302	0.0858358
FSUGold4	0.0830284	0.0820658	0.0827046	0.0817757
IU FSUGold	0.0914953	0.0912007	0.0902246	0.0896274
XS	0.0772768	0.0754875	0.0775578	0.0760218
TM1	0.0939003	0.09369	0.0946969	0.0947533

## Data Availability

Data are contained within the article.

## Appendix A. Selection Tools for the Regression Function and Classical Models

Regression analysis is a valuable method for estimating the relationships between a dependent random variable *Y* (response) and independent variables $X_{1},X_{2},\;\ldots \;X_{k}$ (factors). Let the regression model have the form

(A1) $$\begin{array}{cc} \;\;\;\;\;\;\;Y & =E[Y|X_{1},X_{2},\ldots ,X_{k}]+E\\ \; & =\alpha_{0}+\alpha_{1}\;X_{1}+\alpha_{2}\;X_{2}+\ldots +\alpha_{k}\;X_{k}+E\;, \end{array}$$

where *E* denotes the random error, and $E[Y|X_{1},X_{2},\ldots ,X_{k}]$ is the conditional expectation value of *Y*, and $\alpha_{i}$, i=0,1,2,…,k are the structural parameters. The parameter $\alpha_{0}$ is called the intercept. Considering a sample of *N* models chosen randomly from a population of models, the regression model (A1) can be estimated by

(A2) $$\begin{array}{c} Y={\hat{\alpha }}_{0}+{\hat{\alpha }}_{1}\;X_{1}+{\hat{\alpha }}_{2}\;X_{2}+\ldots +{\hat{\alpha }}_{k}\;X_{k}+\hat{E}\;. \end{array}$$

Here, ${\hat{\alpha }}_{0}$, ${\hat{\alpha }}_{1}$, ${\hat{\alpha }}_{2},\ldots ,{\hat{\alpha }}_{k}$ are the estimators of the structural parameters $\alpha_{0},\alpha_{1},\alpha_{2},\ldots ,\alpha_{k}$ of a particular regression model, and $\hat{E}$ is the estimator of the error term *E* in Equation (A1). The error term $\hat{E}$ variance is denoted by MSE (mean squared error) throughout this paper.

Given a linear regression model with *k* factors, the null hypothesis

(A3) $$\begin{array}{c} H_{0}:\alpha_{1}=\alpha_{2}=\ldots =\alpha_{k}=0\;, \end{array}$$

is a question about the irrelevance of the correlation between the dependent variable *Y* and the group of independent variables $X_{i}$, i=1,2,…,k.

### Appendix A.1. The Characteristics of the Regressions: Coefficient of Determination R^2^ and Adjusted $R_{\mathrm{Adj}}^{2}$

In statistics, the sums of squares, which are used in a regression analysis, measure the variability in data. They reveal the dispersion of data points concerning the mean and how much the response variable differs from the predicted values. For a given dependent variable *Y*, it is convenient to define the total sum of squares as $SSY=\sum_{i=1}^{N}{\left(Y_{i}-\bar{Y}\right)}^{2}$, which is the sum of squares of deviations of the observed $Y_{i}$ from their mean $\bar{Y}$. SSY is often partitioned to the sum of squares due to regression, $SSR=\sum_{i=1}^{N}{\left({\hat{Y}}_{i}-\bar{Y}\right)}^{2}$, and due to error, $SSE=\sum_{i=1}^{N}{\left(Y_{i}-{\hat{Y}}_{i}\right)}^{2}$, where SSE is called the residual (error) sum of squares. This leads to the ANOVA equation for the linear regression, SSY=SSR+SSE [10]. The characteristics of the regressions in use are the mean square due to regression $MSR=SSR/df_{SSR}$, the mean squared error $MSE=SSE/df_{SSE}$, and the coefficient of determination:

(A4) $$\begin{array}{c} R^{2}=\frac{SSR}{SSY}=1-\frac{SSE}{SSY}\in \langle 0,1\rangle \;, \end{array}$$

which measures the ratio of the variability of the dependent variable explained by the regression to the overall variability of this variable. Here, $df_{SSR}=k$ and $df_{SSE}=N-k-1$ are the number of degrees of freedom for SSR and SSE, respectively. The descriptive limits of the correlation strength in this paper are assumed to be $0.1<R^{2}<0.25$ for weak correlation and $R^{2}\geq 0.64$ (|R|≥0.8) for strong correlation. In one-dimensional linear regression $Y=a+b\;X+\hat{E}$, the sign of the Pearson linear correlation coefficient $r_{YX}$ between *Y* and *X*, ref. [10] equals the sign of *b*, $r_{YX}=\mathrm{sgn}\left(b\right)\;\left|R\right|$. An additional statistic to the coefficient of determination $R^{2}$, which considers the number of parameters in the model, is the adjusted coefficient of determination $R_{adj}^{2}$, defined as [51]

(A5) $$\begin{array}{c} R_{adj}^{2}=1-\frac{N-i}{SSY}\;MSE=1-\frac{N-i}{N-K}\left(1-R^{2}\right)\;, \end{array}$$

where the mean square error $MSE=\frac{SSE}{N-K}$ and *N* is the number of observations used to match the model, *K* is the number of parameters in the model, including the intercept, K=k+1, and *i* equals 1 when the model has an offset (intercept) and 0 otherwise. $R_{adj}^{2}$ starts decreasing when the model has too many parameters. The moment in which $R_{adj}^{2}$ starts to drop is a signal that the model no longer needs to be developed.

### Appendix A.2. The Backward Selection Method

#### Appendix A.2.1. *F_partial_* Statistics

One might be tempted to compare models with different numbers of parameters. To this aim, a convenient notation is $SSE_{k}\equiv SSE(X_{1},X_{2},$$\ldots ,X_{k})$. Let the model include $k^{\prime }$ factors $X_{1}$, $X_{2}$, …, $X_{k}$, $X_{k^{\prime }}$, $\;k^{\prime }=k+1.$ To determine the significance of introducing an additional factor $X_{k^{\prime }}$, the partial $F_{p}$ statistics (p for “partial”) for the models ($X_{1},X_{2},\ldots ,X_{k},X_{k^{\prime }}$) and ($X_{1},X_{2},\ldots ,X_{k}$) can be introduced [10]:

(A6) $$\begin{array}{ccc} F_{p} & = & \frac{\left(SSR_{k^{\prime }}-SSR_{k}\right)/\left(df_{SSR_{k^{\prime }}}-df_{SSR_{k}}\right)}{MSE_{k^{\prime }}}\;, \end{array}$$

where, according to the introduced notation, $MSE_{k^{\prime }}=SSE_{k^{\prime }}/df_{SSE_{k^{\prime }}}.$ The statistic $F_{p}$ is a random variable on the sample space, with the F distribution $F_{k^{\prime }-k,N-k^{\prime }-1}$ with $k^{\prime }-k=df_{SSR_{k^{\prime }}}-df_{SSR_{k}}$ and $N-k^{\prime }-1=df_{SSE_{k^{\prime }}}$. For a particular sample, $F_{p}$ takes the observed value $F_{p}^{obs}$. In this case, the empirical significance level (the *p*-value) can be calculated:

(A7) $$\begin{array}{c} p=\mathrm{Prob}(F_{p}\geq F_{p}^{obs})\;. \end{array}$$

If, in the observed sample, for a chosen significance level α, p>α, then there is no reason to reject the null hypothesis $H_{0}:\alpha_{k^{\prime }}=0$, which now reads: “the lower model fits the observed data as well as the higher model”, i.e., the sample gives no incentives to extend the model. Here, the adjectives “lower” and “higher” reflect the number of factors. The lower model is rejected if p≤α. With the hierarchical development of the regression model in this paper, the value of the partial statistics $F_{p}$ in the observed sample and the corresponding *p*-values, (Equation (A7)) for the factor added last can be determined at all stages of model construction. It can be shown that the $F_{p}$ test (A6) for the significance of a one-variable extension of a model with *k* variables coincides with the Student’s *t*-test for the null hypothesis for the structural parameter $\alpha_{k^{\prime }}=0$ (the *p*-values of the tests are the same).

#### Appendix A.2.2. Backward Elimination Method

The backward elimination regression method is a statistical procedure of the model selection used to reduce the number of less significant variables [10]. The selection should start with a possible most complete model and simplify it until it turns out that all the remaining variables have a substantial impact on the accuracy of fitting the model regression to empirical data. As a tool, the partial $F_{p}$ value (A6) (or the empirical significance level, i.e., the *p*-value (A7)), for each variable in the model is calculated. When comparing the highest value of the empirical significance level *p* with the value of the previously chosen significance level α (for the variable to remain in the model, e.g., α=0.01, 0.05), it is possible to decide whether to remove or keep the considered variable. This procedure can be repeated after deciding to neglect a given variable until a model is obtained where all the values of the estimators of the model’s structural parameters are statistically significant.
